# Notes and News

**Published:** 1897-12-25

**Authors:** 


### Notes and News.

(Collected and Communicated.)

The Mercers' Company have voted a sum of 1,000 guineas to the University of Cambridge for the re-building fund of the School of Medicine and Surgery of the University.

The Freemasons are taking a great deal of interest in Guy's Hospital, with the result that they have decided to endow and at once throw open a bed for the use of Masons at the hospital.

A new Eye, Ear, and Throat Hospital has been established at Cork. The opening ceremony was per-ormed by the Mayor, who was supported by a very large company of distinguished visitors.

Mr. T. Backhouse Hulke, F.R.C.S.Eng., has been appointed surgical registrar to the Middlesex Hospital, as successor to Mr. T. H. Kellock, F.R.C.S.Eng., appointed assistant surgeon.

The new wards in the Aberdeen Royal Infirmary have just been renamed. One has been named after Princess Henry of Battenberg, and another after Miss Lumsden, for so many years honorary superintendent of the Children's Hospital, Aberdeen.

The Committee of the North London Hospital for Consumption appeal for funds to enable them to carry on the work. The finances of the hospital are in a most unsatisfactory condition, and there is no endowment whatever. 441 in-patients and 3,420 out-patients were treated last year. New annual subscribers are greatly needed. Contributions will be most thankfully received by L. F. Hill, M.A., hon. secretary, at the offices, 41, Fitzroy Square, W.

The Fishmongers' Company have sent a cheque for £1,000 to the Prince of Wales's Fund, with the following letter: "It is with great pleasure I have to inform you that the Court of the Fishmongers' Company have agreed to contribute £1,000 per annum for five years to His Royal Highness the Prince of Wales's Hospital Fund for London. I beg to enclose herewith a cheque for £1,000, being the first contribution. Will you please sign and return the accompanying receipt?—I remain, yours faithfully, J. Wrench Towse."

Mr. W. J. Morton, chief clerk of the British Home for Incurables, has been appointed assistant secretary of the North London Hospital for Consumption.

We learn that the Clothworkers' Company have voted a munificent annual subscription of £1,000 to the Prince of Wales's Fund "until further order."

Sir Squire Bancroft has promised to give a reading in aid of the Home and Hospital for Jewish Incurables at St. Martin's Town Hall, on January 15th, at which the Chief Rabbi will preside.

The distribution of America's contribution to the Queen's Jubilee is now completed. By it the Prince of Wales's Hospital Fund receives £359, the sum which remained over after the endowment of a bed at Guy's Hospital, Charing Cross Hospital, the London Hospital, and the Seamen's Hospital respectively. Messrs. J. S. Morgan and Co. have handed over the sums contributed to the hospitals and the Fund.

The City of London Hospital for Diseases of the Chest is making a plucky attempt to retrieve its ill-fortunes and secure prosperity once more. The attempt has not been made too soon. Already 120 beds have been closed for want of funds. Now this accommodation is most sadly wanted in the district in which the hospital is situated. We congratulate the designer of the little diagram which forms part of the appeal now being issued. He demonstrates by it in a forcible manner how poor and populous is the area which surrounds the institution. Clearly the means of support cannot come from within the region in which the hospital is situated, and yet the need of its existence is there only too clearly seen. We earnestly hope the response to the present appeal will be ample to revive the good work once more, and enable the committee to open the doors wider to the crowd of disappointed poor whose crowded dwellings are so fatal in cases of diseases of the chest.

A definite agreement has at last been arrived at between Mr. Hooley and the Nottingham Hospital Saturday Committee, respecting his gift to the Nottingham General Hospital. In commemoration of the Queen's Jubilee Mr. E. T. Hooley some months ago offered to subscribe to the Nottingham General Hospital one shilling for every shilling contributed by working men up to the extent of £10,000. Nearly the whole amount was publicly subscribed. Mr. Hooley paid £2,000 on account, but declined any further contribution on the ground that the money had not been given *bona fide* by working men, but to a large extent by employers and others, who were not contemplated under the terms of the original offer. The matter has given rise to much discussion locally, and prolonged negotiations have taken place between Mr. Hooley and the Hospital Saturday Committee. The result was that at his request the lists were analysed, he undertaking to abide by the decision of the committee as to what were legitimate contributions of working men. It was reported at a meeting on Saturday night that these had been assessed at £5,220, and Mr. Hooley had given a cheque for the further amount thus shown to be due from him in addition to the £2,000 already paid. It was also stated that he had placed an order with his banker for payment of the balance up to £10,000, if the amount could be raised by working men only.

